# Psilocin glucuronide in whole blood: a stable and useful biomarker of psilocybin intake

**DOI:** 10.1093/jat/bkag015

**Published:** 2026-02-22

**Authors:** Marianne Skov-Skov Bergh, Inger Lise Bogen, Merete Vevelstad, Åse Marit Leere Øiestad

**Affiliations:** Division of Laboratory Medicine, Department of Forensic Sciences, Oslo University Hospital, Oslo, Norway; Division of Laboratory Medicine, Department of Forensic Sciences, Oslo University Hospital, Oslo, Norway; Department of Pharmacy, The Faculty of Mathematics and Natural Sciences, University of Oslo, Oslo, Norway; Division of Laboratory Medicine, Department of Forensic Sciences, Oslo University Hospital, Oslo, Norway; Division of Laboratory Medicine, Department of Forensic Sciences, Oslo University Hospital, Oslo, Norway

## Abstract

Detecting psilocybin use is challenging because it rapidly converts to its psychoactive metabolite psilocin, and both compounds are unstable in blood. Bufotenin, a structural isomer of psilocin, may exhibit comparable instability in blood. For reliable detection, we developed and validated an LC-MS/MS method to simultaneously quantify psilocin, bufotenin, and their metabolites, psilocin glucuronide (PSG) and 5-hydroxyindole-3-acetic acid (5-HIAA), in human whole blood. We prepared blood samples by protein precipitation and lipid removal filtration. Analytes were separated using a biphenyl column. The method was validated according to AAFS guidelines with LOQs of 2.4 nM for psilocin, PSG, and bufotenin, and 30 nM for 5-HIAA. We assessed analyte stability in whole blood under conditions relevant to forensic sample handling. Psilocin degraded by 46%–66% at room temperature and 66%–76% at 4°C after one day, increasing to 88%–99% and 94%–100% after three days. At −20°C, degradation slowed, with up to 51% loss after one month and ≥91% after three months. In contrast, PSG remained stable for 14 days at both room temperature and 4°C, and for at least one year at −20°C, making it a reliable biomarker of psilocybin intake. Bufotenin showed moderate stability, while 5-HIAA was unsuitable as a biomarker due to its endogenous presence. Our method enables direct quantification of PSG, offering a straightforward and accurate alternative to indirect approaches. We demonstrated the method’s applicability by analyzing 23 forensic blood samples that screened positive for psilocin or PSG, with PSG quantified in nearly all cases, even when psilocin was below LOQ. These findings confirm PSG as a specific and stable biomarker of psilocybin use, and its integration into routine forensic workflows could significantly improve detection reliability.

## Introduction

**Figure 1:**
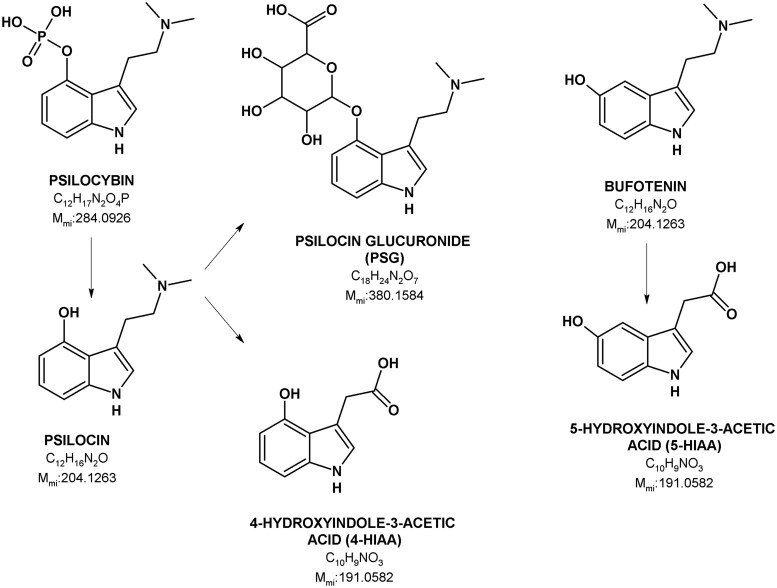
Structural formulas and monoisotopic mass (m_mi_) of psilocybin, psilocin, PSG, 4-HIAA, bufotenin, and 5-HIAA.

Psilocybin (*O*-phosphoryl-4-hydroxy-*N*, *N*-dimethyltryptamine) and psilocin (4-hydroxy-*N*, *N*-dimethyltryptamine) are the main psychoactive compounds found in hallucinogenic mushrooms of the genus *Psilocybe*, commonly referred to as “magic mushrooms” (Figure 1) [1]. Psilocin is a potent 5-HT_2A_ receptor agonist and the primary compound responsible for the psychedelic effects of *Psilocybe* mushrooms [2, 3]. After oral intake, psilocybin acts as a prodrug and is rapidly dephosphorylated to psilocin in the stomach and intestines before entering systemic circulation [3, 4]. Bufotenin (5-hydroxy-*N*, *N*-dimethyltryptamine) is a positional isomer of psilocin (Figure 1). It is found in certain psychedelic mushrooms (*Amanita muscaria*), in the skin glands of Brazilian *Rhinella* toads, and plants of the Leguminosae family [5]. Additionally, endogenous bufotenin has been reported in human urine and stool [6]. Although bufotenin and psilocin are structurally similar, it is debated whether bufotenin has hallucinogenic effects [7–9].

Psilocybin, psilocin, and bufotenin are controlled substances in countries such as Norway, the United Kingdom, and the United States [10–12]. Yet, 3750 seizures of hallucinogenic mushrooms, totaling 333 kg, were reported across 24 countries in Europe in 2023 [13]. Seizures in Norway increased in 2020, but later stabilized [14], while inquiries to the Norwegian Poison Information Centre regarding human exposure to psilocin-containing mushrooms have steadily increased since 2019 (Anita von Krogh, Norwegian Poison Information Centre, personal communication, 2025). In the US, seizures of hallucinogenic mushrooms surged between 2017 and 2022, likely reflecting increased interest in their therapeutic potential for psychiatric conditions [15]. The determination of these compounds in biological samples are thus of interest in forensic toxicology laboratories.

Quantitative analysis of psilocybin, psilocin, and bufotenin in blood can be challenging. Since psilocybin is rapidly metabolized pre-systemically to psilocin, and is highly unstable in blood samples, psilocin is commonly measured as a proxy for psilocybin intake [2, 3, 16, 17]. However, psilocin is also known to be highly unstable in blood and in neat solutions, and is prone to oxidation when exposed to air, light, and alkaline pH [5, 18–21]. Given the structural similarities, this might also be the case for bufotenin. Additionally, pre-analytical sample handling may vary considerably, with samples being exposed to room temperature for extended periods. In Norway, this poses a particular challenge, as only two forensic toxicology laboratories serve the entire country. This results in long transport distances and increasing delays due to slower postal services. In forensic casework, ingested psilocin or bufotenin may go undetected due to pre-analytical degradation. This emphasizes the importance of targeting more stable metabolites to confirm intake.

*In vivo*, the elimination half-life of psilocin in human plasma is 1.8–4.7 hours [2, 3, 22, 23] following oral administration of psilocybin, and 1.2 hours [3, 23] after intravenous administration. Psilocin is mainly metabolized to pharmacologically inactive psilocin-*O*-glucuronide (PSG) and 4-hydroxyindole-3-acetic acid (4-HIAA; Figure 1) [2, 3, 19, 24–26]. Of the two psilocin metabolites, PSG has a significantly longer half-life (3.8–4.7 hours) than 4-HIAA in humans (1.6–2.4 hours), remaining in the body for a longer time period after oral administration [2, 3, 24]. *In vitro* studies have reported that PSG is more stable than psilocin in whole blood [19]. Bufotenin is rapidly metabolized in humans, with approximately 90% of the compound eliminated within 12 hours [27]. Like serotonin, bufotenin is primarily metabolized to 5-hydroxyindole-3-acetic acid (5-HIAA, Figure 1), which has a serum half-life of 1.3 hours in humans [27–29].

In countries that have established legal drug concentration limits for assessing impairment, such as Norway, these thresholds are typically defined for whole blood [30]. Based on the available literature, no bioanalytical methods have been developed for the simultaneous determination of psilocin, bufotenin, and their major metabolites in human whole blood. Existing methods for psilocin and its metabolites in human blood were developed for plasma or serum [2, 16, 19, 23, 24, 31–33], and to the best of our knowledge, no methods have been developed for the simultaneous determination of bufotenin and 5-HIAA in human blood. As far as we are aware, no validated method directly quantify PSG levels in biological samples against a reference standard. Existing approaches involve enzymatic deglucuronidation of PSG to psilocin prior to analysis, with PSG levels calculated as the difference between deglucuronidated and non-deglucuronidated samples [16, 24, 31–36]. Depending on the efficacy of the enzymatic reaction, this may only provide an estimate of the PSG concentration.

In this study, we developed and fully validated a liquid chromatography tandem mass spectrometry (LC-MS/MS) method for the determination of psilocin, PSG, bufotenin, and 5-HIAA in human whole blood. Due to the differences in pre-analytical handling of samples in forensic toxicology laboratories, we also performed comprehensive stability studies of the analytes in whole blood at different storage conditions. The applicability of the method and usefulness of PSG as a marker of psilocybin intake was demonstrated by quantitating psilocin and PSG in whole blood samples from forensic toxicology cases.

## Material and methods

### Chemicals, reagents and materials

Psilocin, psilocin-d_6_, and bufotenin-d_6_ were obtained from Chiron AS (Trondheim, Norway). Bufotenin hydrochloride was obtained from Cayman Chemical (Ann Arbor, MI, USA). Psilocin-O-β-D-glucuronide was obtained from Alsachim (Strasbourg, France). Buprenorphine-3-β-D-glucuronide-d_4_ was obtained from Cerilliant Corporation (Round Rock, TX, USA). Ascorbic acid (AA) and 5-hydroxyindole-3-acetic acid were obtained from Sigma Aldrich (St. Louis, MO, USA). 5-Hydroxyindole-3-acetic acid-d_5_ was obtained from CDN isotopes (Quebec, Canada). Methanol (MeOH, Chromasolv^®^) was obtained from Honeywell/Riedel-de Haën (Seelze, Germany). Acetonitrile (ACN) was obtained from J.T.Baker (Center Valley, PA, USA). Ethanol (EtOH) was obtained from Merck Millipore (Billerica, MA, USA). Formic acid (FA) and ammonium formate were obtained from VWR (Radnor, PA, USA), Type 1 water (18.2 MΩ) purified with a Synthesis A 10 milli-Q system from Millipore (Billerica, MA, USA) was used. Blank human whole blood used for calibrators and quality control (QC) samples was obtained from the Blood Bank at Oslo University Hospital (Oslo, Norway). The blood was supplemented with heparin (final concentration: 17 IU/mL; Leo Pharma, Lysaker, Norway) as an anticoagulant and sodium fluoride (final concentration: 4 mg/mL, Merck KGaA, Darmstadt, Germany) as a preservative.

### Preparation of calibrators, QC samples, and internal standards

Mixed stock solutions were prepared separately for all analytes and for all internal standards (ISs) in a vol/vol mixture of 96% ACN: H_2_O (1:1) and 4% saturated AA in EtOH: H_2_O (30:70), hereafter referred to as “the AA solution.” AA was used because it can prevent the oxidation of phenolic tryptamines, such as psilocin, bufotenin, and 5-HIAA, during storage and sample preparation [7, 21, 23, 37]. Working solutions for seven calibrators and six quality control (QC) samples were prepared by diluting the mixed stock solution in the AA solution. Calibrators (1–500 nM) and QC samples (1.2–400 nM) were freshly prepared prior to each analysis by adding working solution (50 µL) to human whole blood (100 µL). A working solution of the ISs (500 nM) was prepared by diluting the mixed stock solution with AA solution. Due to the lack of an isotope-labeled internal standard for PSG, buprenorphine glucuronide-d4 was used as the IS.

### Sample preparation

Whole blood samples (100 µL) were transferred to plastic tubes and AA solution (50 µL) was added before vortexing. The IS mixture (25 µL) was added to the tubes followed by vortexing. Ice-cold ACN: MeOH mixture (400 µL, 85:15, v/v) was added to all samples before 30 s of vortexing and 10 min centrifugation at 4°C and 5200 g. Captiva EMR lipid removal cartridges (Agilent, CA, USA) were prepared by adding 200 µL of a vol/vol mixture of ACN:H_2_O (4:1, v/v) with 1% FA (v/v, of total volume) to each ­cartridge. The supernatants were added to the cartridges, left to set for 2 min, before the filtrate was eluted using a vacuum manifold. The samples were evaporated to dryness at 40°C under a stream of N_2_ and reconstituted in 50 µL 25 mM FA added 2.5% MeOH and 4% saturated AA in EtOH:H_2_O (30:70). The samples were centrifuged at 4°C and 5200 g for 5 min and the supernatants were transferred to autosampler vials before LC-MS/MS analysis.

### Optimization of sample preparation procedure

The effect of light exposure on analyte stability during sample preparation was examined by fortifying water (100 µL, *n* = 8) with the highest calibrator (final conc. 500 nM, 50 µL), adding protein precipitation reagent (400 µL, ACN:MeOH, (85:15, v/v)) and solvent for lipid removal cartridge preparation (200 µL, ACN:H_2_O (4:1, v/v) with 1% FA (v/v, of total volume) before placing samples in daylight (*n* = 4) or darkness (*n* = 4) for three hours. All samples were diluted 1:4 with 25 mM FA before LC-MS/MS analysis.

The effect of temperature and time on analyte stability during evaporation was examined by fortifying water (100 µL, *n* = 24) with the highest calibrator (final conc. 500 nM, 50 µL), then adding protein precipitation reagent (400 µL, ACN:MeOH, 85:15) and solvent for lipid removal cartridge preparation (200 µL, ACN:H_2_O (4:1, v/v) with 1% FA (v/v, of total volume) before evaporation in a N_2_ evaporator. The mixtures were evaporated to dryness, or to dryness plus an additional 20 min, at 30, 40, 50, and 60°C, with three replicates (*n* = 3) for each temperature and time point. The samples were reconstituted in a mixture of 25 mM FA, 2.5% MeOH and 4% AA (50 µL) and centrifuged at 4°C and 5200 g for 5 min. The supernatants were transferred to autosampler vials before LC-MS/MS analysis.

### LC-MS/MS analysis

Whole blood samples were analyzed with an Acquity UPLC™ system (Waters, Milford, MA, USA) coupled to an Xevo-TQS triple quadrupole MS with an electrospray ionization interface (Waters).

When developing the LC method, we used a mobile phase consisting of 25 mM FA or 10 mM ammonium formate (pH 3.1) and MeOH to examine three different columns: Acquity HSS T3 (2.1 × 100 mm, 1.8 µm, Waters), Acquity BEH C18 (2.1 × 100 mm, 1.7 µm, Waters), and Kinetex biphenyl (2.1 × 100 mm, 1.7 µm, Phenomenex, Verløse, Denmark). The optimized chromatographic method was achieved using the Kinetex biphenyl column kept at 60°C with a mobile phase consisting of 25 mM FA (solvent A) and MeOH (solvent B) at a flow rate of 0.5 mL/min. The separation was performed using the following gradient profile: 0–1.5 min; 2.5% B, 1.5–3.0 min; 2.5%–10% B, 3.0–3.5 min; 10% B, 3.5–3.6 min; 10%–50% B, 3.6–4.6 min; 50% B, 4.6–4.7 min; 50%–100% B, 4.7–6.7 min; 100% B, 6.7–6.8 min; 100%–2.5% B, 6.8–7.8 min; 2.5% B. The injection volume was 3 µL and the injection technique employed was partial loop with needle overfill.

MS/MS analysis of the analytes was performed with the electrospray in positive ionization mode and the quadrupoles in multiple reaction monitoring (MRM) mode. A capillary voltage of 1 kV, source temperature of 150°C, desolvation gas temperature of 500°C, cone gas flow of 300 L/h, and desolvation gas flow of 1000 L/h were used. Data was acquired and processed using Masslynx™ 4.1 software (Waters). The MRM transitions, MS/MS parameters, ion ratios, and retention times for the analytes are displayed in Table 1.

**Table 1:** MRM transitions, ion ratio, MS/MS parameters, and retention times for the analytes and internal standards.

Analyte	MRM transitions	Ion ratio^a^	MS/MS parameters		Retention time (min)^b^
			Cone voltage (V)	Collision energy (eV)	
Psilocin	**205.1 > 58.0**	0.47	25	15	3.4
	205.1 > 160.1		15	20	
PSG	381.0 > 160.0	0.32	15	20	3.0
	**381.0 > 205.0**		15	20	
Bufotenin	**205.1 > 58.0**	0.77	25	15	2.3
	205.1 > 160.1		15	20	
5-HIAA	192.0 > 91.0	0.06	22	38	3.3
	**192.0 > 146.0**		22	16	
Psilocin-d_6_	**211.1 > 64.1**		15	20	3.4
	211.1 > 160.1		15	20	
Buprenorphine glucuronide-d_4_	**648.3 > 472.3**		64	40	4.4
Bufotenin-d_6_	**211.1 > 64.1**		15	20	2.3
	211.1 > 160.1		15	20	
5-HIAA-d_5_	197.0 > 96.0		22	38	3.3
	**197.0 > 151.0**		22	16	

Transitions used for quantification are written in bold.

a Ion ratios had a relative standard deviation (RSD) ≤6.5%.

b Retention times were stable, varying by ≤0.3%.

### Method validation

We validated the method according to the guideline of the American Academy of Forensic Sciences (AAFS) [38], as described in detail in the Supplementary Material (Suppl.1), assessing the following parameters: calibration model, within-run precision, between-run precision and bias, limit of detection (LOD), limit of quantification (LOQ), interference studies, processed sample stability, recovery, matrix effects (ME), carry-over, and dilution integrity.

### Preparation of fortified whole blood samples for stability studies

In addition to assessing processed-sample stability during method validation, we evaluated analyte stability in fortified whole blood to address pre-analytical handling relevant to forensic toxicology. Analyte stability was assessed in fortified whole blood samples prepared from three blood sources and stored under various conditions. Samples were fortified at concentrations of 30 and 400 nM (*n* = 3 per level). Stability was examined after storage for up to two weeks at room temperature, up to two weeks at 4°C, up to one year at −20°C, and after three freeze/thaw cycles. We considered analytes stable if the deviation from the initial concentration was ≤ ±20%.

### Forensic case samples

At the Department of Forensic Sciences, Oslo University Hospital, antemortem whole blood samples from drivers suspected of driving under the influence of drugs or alcohol (DUID), as well as other criminal cases, are routinely screened for psilocin. Following initial findings in this study, screening was expanded to include psilocin glucuronide (PSG). Between 1 January and 31 December 2024, we analyzed a selection of blood samples that tested positive for psilocin or PSG during routine screening, using the developed LC-MS/MS method. The concentrations of psilocin and PSG quantified in these samples (*n* = 23) were later retrieved anonymously from the Laboratory Information Management System (LIMS) database at Oslo University Hospital.

## Results and discussion

### Optimization of sample preparation

Since psilocin is known to be sensitive to light [18], we investigated whether light exposure during sample preparation affected analyte stability. To assess this, water samples were fortified with analytes and supplemented with all reagents typically used during sample preparation. The samples were then stored either in daylight or in darkness. The deviation in peak height between the two groups was ≤ ±20% for all analytes, indicating that light exposure did not significantly affect their stability during sample preparation (Suppl. 2). Consequently, protection from light during sample preparation was deemed unnecessary.

Ensuring the stability of psilocin during the evaporation step of sample preparation is crucial [21]. Therefore, we examined the effects of temperature and evaporation time on analyte stability to determine the optimal parameters for this step (Suppl. 3). Psilocin was the analyte most affected by temperature, displaying the highest stability at 40°C. Stability dramatically decreased at temperatures above 40°C and was also lower at 30°C, likely due to the increased evaporation time. In contrast, PSG remained highly stable across all tested conditions, showing minimal variation compared to psilocin. We also observed that the peak height of psilocin varied considerably between samples (RSD 36%–195%), which was most likely due to the analyte’s unstable nature. The psilocin IS, psilocin-d_6_, showed similar results regarding stability and variation in peak height (RSD 35%–196%). Since psilocin was most stable at 40°C and the other analytes showed good stability at this temperature (76%–88%), 40°C was used in the final sample preparation procedure. Extending the evaporation time by 20 min significantly reduced psilocin stability, resulting in a 91% decrease in peak height at 40°C compared to the standard time. In comparison, bufotenin and 5-HIAA showed smaller reductions of 29% and 43%, respectively. To minimize degradation, we ensured that the samples were removed from the evaporator as quickly as possible after being evaporated to dryness.

### Chromatographic separation

**Figure 2:**
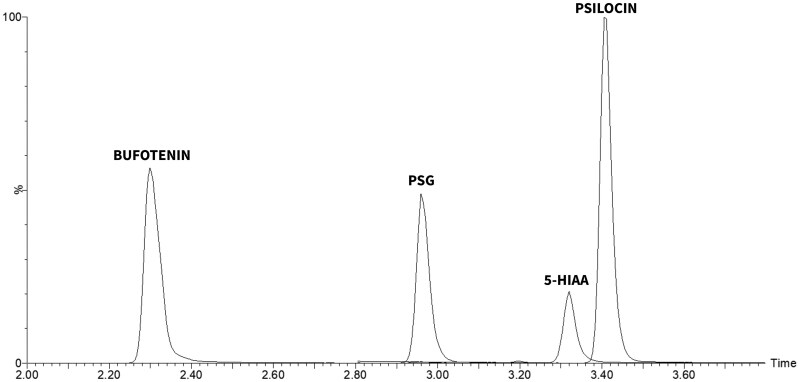
Optimized separation of 500 nM psilocin, PSG, bufotenin, and 5-HIAA in a prepared blood sample on a Kinetex biphenyl column using a mobile phase consisting of 25 mM formic acid and methanol. The separation was performed as described in the Method section “LC-MS/MS analysis”.

During LC method development, we examined three different columns together with two mobile phase additives (formic acid or ammonium formate) in combination with methanol, as described in the “LC-MS/MS Analysis” section (data not shown). The two isomers psilocin and bufotenin had good separation on all combinations examined. However, bufotenin and PSG co-eluted or had limited separation on all columns when using ammonium formate buffer as an additive. Employing FA in the mobile phase resulted in baseline separation across all tested columns. Among these, the Kinetex biphenyl column provided the best resolution and selectivity and was therefore chosen for the final method. A chromatogram obtained using the optimized gradient profile is displayed in Figure 2.

### Method validation

Calibration curves for all four analytes were linear, with R^2^ ≥ 0.99 (Table 2). The LOD and LOQ were 1.2 nM and 2.4 nM for all compounds except 5-HIAA. Since 5-HIAA is also endogenously produced, its exact LOD could not be determined. The LOQ was 2.4 nM for psilocin, PSG, and bufotenin. For 5-HIAA, the LOQ was set at 30 nM and considered semi-quantitative because the coefficient of variation (CV) was 23% (Table 2). The elevated LOQ likely resulted from the endogenous presence of 5-HIAA in the blood samples used for validation. Chromatograms at LOD and LOQ are provided in the Supplementary material (Suppl. 4 and 5).

**Table 2:** Validation parameters including calibration model (linear range, R²), LOD, nominal QC concentrations and LOQ, within-run precision, between-run precision and accuracy, matrix effects (ME), recovery following protein precipitation (PPT), filtration and evaporation, and recovery after evaporation only.

Analyte	Calibration model		LOD, nM (ng/mL)	Nominal QC conc., nM (ng/mL)	Within-run precision	Between-run precision and accuracy		Matrix effects			Recovery			
	Linear range, nM (ng/mL)	R^2^			CV (%)	CV (%)	Bias (%)	ME (%)	CV (%)	ME corr. (%)^a^	PPT+filtration+evaporation (%)	CV (%)	Evaporation (%)	CV (%)
Psilocin	2.0–500 (0.4–102)	0.9981	1.2 (0.25)	**2.4 (0.5)**	14	15	−17							
				6.0 (1.2)	3.9	5.4	7.8							
				30 (6.1)	3.7	6.2	6.1	114	12	101	20	20	66	3.9
				200 (41)	4.6	3.7	−2.3							
				400 (82)	7.8	8.2	5.7	109	16	99	22	9.5	69	2.9
PSG	2.0–500 (0.8–190)	0.9893	1.2 (0.5)	**2.4 (0.9)**	11	5.2	−0.5							
				6.0 (2.3)	12	6.1	2.3							
				30 (11)	13	8.0	4.0	115	13	111	33	14	92	8.4
				200 (76)	5.3	5.7	−2.7							
				400 (152)	7.1	6.3	5.5	110	17	89	44	8.8	95	5.1
Bufotenin	1.0–500 (0.2–102)	0.9925	1.2 (0.5)	**2.4 (0.5)**	14	9.0	−19							
				6.0 (1.2)	8.6	5.2	4.4							
				30 (6.1)	5.0	5.0	9.4	119	11	96	50	13	97	1.5
				200 (41)	4.9	4.0	2.5							
				400 (82)	4.9	5.3	5.1	108	15	96	56	5.9	94	2.1
5-HIAA^b^	25–500 (4.8–96)	0.9980	–	**30^c^ (5.7)**	29	23	5.6	340	26	298	99	34	94	17
				200 (38)	11	9.9	−9.3							
				400 (76)	7.4	9.5	0.1	109	11	103	50	7.8	93	2.9

Concentrations are reported in nM, with ng/mL values in parentheses. Nominal QC concentrations corresponding to the LOQ are indicated in bold. Calibration parameters (linear range and R^2^) were derived from five calibration curves (*n* = 5). LOD was determined from triplicate QC samples analyzed over three runs (*n* = 9), and LOQ from triplicate QC samples over five runs (*n* = 15). Within-run precision and between-run precision and accuracy were assessed using triplicate QC samples (*n* = 3) per concentration in each run. Five runs were performed for 2.4, 6.0, and 30 nM, and six runs for 200 and 400 nM.

a Matrix effects corrected with internal standard.

b 5-HIAA is endogenous, present in all samples before fortification, and can also be detected in blank samples with an S/N > 3.3. LOD could thus not be determined, and recovery and ME at the low QC concentration (30 nM) may be erroneous.

c %CV exceeds 20%, which suggests that the actual LOQ is likely higher than indicated here.

Within-run precision, as well as between-run precision and accuracy, were acceptable for all analytes (Table 2), except for 5-HIAA at LOQ (CV= 29 and 23%, respectively). The higher CV was most likely due to 5-HIAA being endogenous and present in blank blood at variable amounts. Recovery after protein precipitation and filtration was 29%–52% for psilocin, PSG, and bufotenin. 5-HIAA displayed a recovery of 100 and 50% at 30 and 400 nM, respectively. The high recovery observed at the lower concentration is most likely due to the presence of endogenous 5-HIAA in whole blood, which artificially elevated the measured concentration. The recovery after evaporation was similar for all compounds (92%–97%), except for psilocin, where the recovery decreased by 31%–34% during evaporation (Table 2), in line with our observations described in “Optimization of Sample Preparation Procedure.” We used lipid removal cartridges primarily to prevent phospholipid-related contamination of the LC–MS/MS system. Although this step may have reduced analyte recoveries, maintaining method robustness for routine forensic casework was considered a higher priority. ME was observed for 5-HIAA, but only at the lowest QC concentration (Table 2). We observed no carry-over for the analytes. When examining dilution integrity, all analytes displayed acceptable within-run precision, as well as between-run precision and accuracy (data not shown).

Interference from blank blood matrix, ISs, and commonly encountered drugs and pharmaceuticals was evaluated. No interfering peaks were observed at or near the retention times of psilocin and PSG. For bufotenin, an interfering peak from the blood matrix eluted close to the analyte for the secondary MRM transition. This caused greater variability in the ion ratio for bufotenin, particularly at lower concentrations. Due to the endogenous presence of 5-HIAA, interferences were not evaluated for this compound.

### Stability of analytes in fortified blood samples

**Figure 3:**
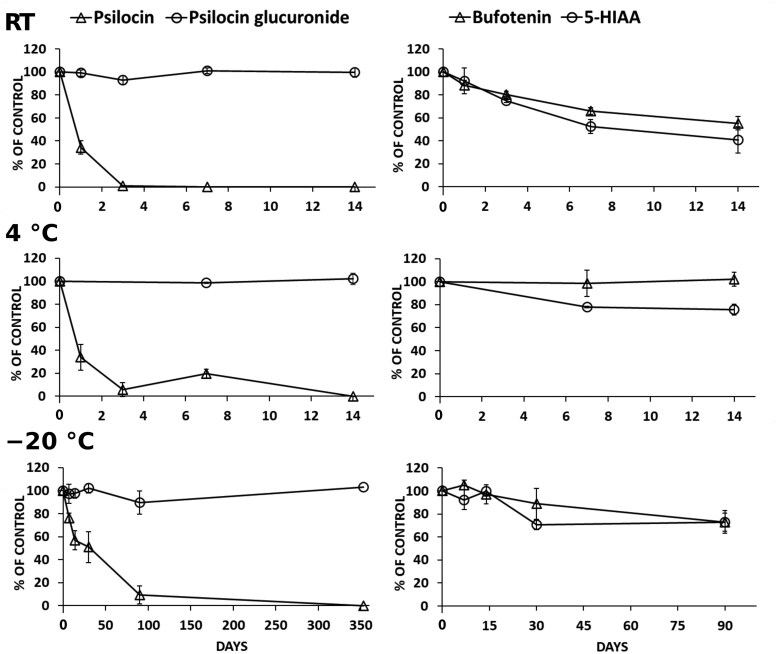
Stability of the analytes in fortified whole blood (final conc. 400 nM) at different storage conditions. Data are presented as mean ± SD (*n* = 3), except for the 1-year time point (*n* = 1). RT, room temperature.

Pre-analytical handling of blood samples varies widely among forensic toxicology laboratories, with samples often exposed to different temperatures for extended time periods. To adress this, we performed an extensive stability study of the analytes in human whole blood. Previous whole-blood stability studies have used sodium fluoride as a preservative [19, 21]. In the present work, stability was assessed in heparinized whole blood supplemented with sodium fluoride, reflecting our routine pre-analytical conditions. Samples were stored at room temperature and 4°C for up to 14 days, and at −20°C for up to one year. Stability results for analytes in whole blood are shown in Figure 3 and detailed in Supplementary material (Suppl. 6). We also examined analyte stability in whole blood after three freeze/thaw cycles (Suppl. 6).

Psilocin was unstable in fortified whole blood under all storage conditions tested. At room temperature, 46%–66% degraded after one day, 88%–99% after three days, and the compound was completely lost after one week. At 4°C, psilocin degraded by 66%–76% after one day, 94%–100% after three days and was completely lost after one week. These findings are concerning, as forensic blood samples are often exposed to room temperature for hours or even days before analysis. Blood samples are also commonly stored in the refrigerator while awaiting analysis. This indicates that psilocin in positive samples may completely degrade and become undetectable before analysis. When stored at −20°C, psilocin showed improved stability, with 24%–32% degradation after one week, 27%–43% after two weeks, 49%–51% after one month, and 91%–100% after three months. To maximize the detection window for psilocin, samples should be stored at the lowest possible temperature and analyzed promptly upon arrival. However, because psilocin remained stable for only two freeze/thaw cycles, the number of thaw cycles should be minimized. In contrast, the major psilocin metabolite PSG remained stable for 14 days at room temperature and 4°C, and for up to one year at −20°C, including 3 freeze/thaw cycles (Figure 3 and Suppl. 6).

Our findings align with Martin et al., who studied the short-term stability of psilocin [21] and PSG [19] in human whole blood at room temperature, 4°C, and −20°C for up to one week. However, we did not observe the increased stability of psilocin at 4°C reported by Martin et al., and psilocin degradation at −20°C was slower in our study. The greater stability in plasma at 4°C reported by Martin et al. [19, 21] contrasts with the pronounced instability we observed in whole blood and may indicate that erythrocyte-related processes contribute to degradation during preanalytical handling and storage. Additionally, PSG exhibited greater stability in our study. To our knowledge, no other studies have reported long-term stability of these analytes in human whole blood. To further extend the detection window for psilocin, PSG should be included in forensic toxicology methods to increase the likelihood of detecting psilocybin intake.

Bufotenin remained stable for up to three days at room temperature, degrading gradually by 34% after one week and 45% after two weeks (Figure 3 and Suppl. 6). At 4°C, the compound was stable for one week, with 32% loss after two weeks. At −20°C, changes were minor after one month (22% loss) but increased to 27%–50% after three months. Bufotenin tolerated three freeze/thaw cycles. At the low QC level (30 nM), 5-HIAA concentrations increased markedly (42%–343%), likely due to serotonin degradation generating additional 5-HIAA. This confounding effect means that stability results for 5-HIAA at 30 nM cannot reliably represent the true stability of the metabolite. At the high QC concentration (400 nM), 5-HIAA showed limited stability, remaining stable for one day at room temperature, with minor losses after two to four weeks at 4°C (−22%–24%). The metabolite remained stable for two weeks at −20°C but showed losses at one and three months (−27%–29%). 5-HIAA remained stable through three freeze/thaw cycles. To our knowledge, no previous studies have reported the stability of bufotenin and 5-HIAA in human whole blood. Bufotenin was more stable than psilocin, whereas analyzing 5-HIAA did not extend the detection window. As an endogenous compound, 5-HIAA is not a suitable selective marker for bufotenin, making direct analysis of the parent drug preferable.

In summary, our stability study demonstrated clear differences between the analytes. Psilocin was highly unstable under all tested conditions, degrading rapidly at room temperature and 4°C, and only partially stable at −20°C. In contrast, PSG remained stable for up to one year at −20°C, confirming its value as a reliable biomarker of psilocybin intake. Bufotenin showed moderate stability, while 5-HIAA was influenced by serotonin degradation at low concentrations, limiting its usefulness. Overall, PSG is the most robust analyte for forensic detection, and direct analysis of bufotenin is preferable to its metabolite.

### Stability of analytes in extracted blood samples

We evaluated analyte stability in extracted whole blood under short-term storage conditions (Suppl. 7), following AAFS guidelines [38]. All analytes remained stable for one day at room temperature and seven days at −20°C, except for 5-HIAA at the low QC concentration.

### Strengths and weaknesses of the developed method

Our method achieved a lower LOQ compared to two previously published LC-MS/MS methods for psilocin in whole blood (3.8 nM [17] and 9.8 nM, respectively [39]). The structural isomers psilocin and bufotenin were baseline separated, with retention times differing by over one minute, ensuring unambiguous identification. Although bufotenin was not detected in the analyzed blood samples, it can be formed endogenously from serotonin and has been reported in urine of patients with schizophrenia or autism spectrum disorders [40], which may complicate interpretation in forensic casework. Our method is the first to directly quantify PSG against a reference standard, rather than indirectly by deglucuronidating PSG to psilocin and comparing the psilocin concentrations in deglucuronidated and non-deglucuronidated samples [16, 24, 31–36]. Direct determination of PSG improves robustness and accuracy, because analysis results are not dependent on the efficacy of an enzymatic reaction, which may yield semi-quantitative estimates. The endogenous presence of 5-HIAA in whole blood negatively affected most of the validation parameters. This limitation could have been avoided by using a surrogate matrix, or isotope labeled 5-HIAA as a surrogate analyte, during the validation.

### Evaluation of psilocin glucuronide as a biomarker for psilocybin intake

To assess the method’s applicability and PSG’s potential as a biomarker of psilocybin intake, we analyzed 23 authentic whole blood samples from DUID and other criminal cases in which psilocin and PSG were detected during targeted screening, using the developed LC-MS/MS method (Table 3). Psilocin was above the LOQ in 19 cases (83%), while PSG was above the LOQ in 22 cases (96%). Psilocin concentrations ranged from 3.3 to 134 nM (mean: 23 nM; median: 7.5 nM). Four cases had concentrations below the LOD (0.1–0.8 nM). PSG concentrations ranged from 2.5 to 510 nM (mean: 67 nM; median: 34 nM). PSG was below the LOQ in one sample (2.2 nM). The PSG/psilocin ratio ranged from 0.6 to 11 (mean: 4.4; median: 3.8) in samples with psilocin above the LOQ. In all but one case, the ratio exceeded 1. PSG demonstrated significantly greater stability in whole blood, and previous studies have also reported a longer *in vivo* half-life compared to psilocin [2, 3, 24], both factors reinforcing its suitability as a biomarker. PSG is highly specific to psilocin and unlikely to be formed from other substances. However, since PSG is pharmacologically inactive [3], its presence alone cannot indicate impairment. Nonetheless, detecting PSG in blood confirms psilocybin use and may assist in assessing its potential impact on behavior. In cases where the determination of very low psilocin concentrations is critical for casework, enzymatic deconjugation may offer in­creased sensitivity by enabling quantification of total psilocin. However, this approach requires more extensive sample preparation.

**Table 3:** Concentrations of psilocin and PSG in whole blood samples from DUID and other criminal cases (reported in nM, with corresponding ng/mL values in parentheses).

Sample ID	Psilocin, nM (ng/mL)	PSG, nM (ng/mL)	PSG/psilocin ratio
1	43 (8.8)	79 (30)	1.8
2	134 (27)	510 (194)	3.8
3	<LOQ	<LOQ	-
4	4.8 (1.0)	16 (6.1)	3.2
5	<LOQ	13 (4.9)	-
6	<LOQ	10 (3.8)	-
7	<LOQ	2.5 (0.95)	-
8	10 (2.0)	60 (23)	5.9
9	4.1 (0.8)	23 (8.7)	5.5
10	23 (4.7)	24 (9.1)	1.0
11	7.5 (1.5)	81 (31)	11
12	5.2 (1.1)	2.9 (1.1)	0.6
13	90 (18)	95 (36)	1.1
14	6.3 (1.3)	44 (17)	7.0
15	3.3 (0.7)	6.9 (2.6)	2.1
16	25 (5.1)	64 (24)	2.5
17	44 (9.0)	166 (63)	3.8
18	6.6 (1.3)	22 (8.4)	3.3
19	3.8 (0.8)	16 (6.1)	4.2
20	7.5 (1.5)	58 (22)	7.7
21	9.2 (1.9)	50 (19)	5.4
22	10 (2.0)	112 (43)	11
23	6.3 (1.3)	22 (8.4)	3.5
Mean (nM)^a^	23 (4.7)	67 (26)	4.4
Median (nM)^a^	7.5 (1.5)	34 (13)	3.8
Range (nM)^a^	3.3–134 (0.7–27)	2.5–510 (1.1–194)	0.6–11

Abbreviations: PSG, psilocin glucuronide; DUID, driving under the influence of drugs or alcohol.

a Not including values below LOQ.

The usefulness of 5-HIAA as a marker of bufotenin intake could not be evaluated in forensic samples because no bufotenine-positive samples were available during this study. However, since 5-HIAA is an endogenous compound with baseline levels that elevates the LOQ, it is not an optimal marker and may cause false positives or misinterpretation.

## Conclusion

We developed and validated an LC-MS/MS method for psilocin, bufotenin, and their metabolites PSG and 5-HIAA in human whole blood. The method enables direct quantification of PSG, offering a straightforward and accurate alternative to indirect approaches. Our stability studies confirmed that psilocin degrades rapidly under typical forensic storage conditions, highlighting the need for prompt analysis and low temperature storage to maximize detection likelihood. In contrast, PSG remained stable for at least a year at −20°C, making it a more reliable marker than psilocin for psilocybin intake. Bufotenin showed moderate stability, while its metabolite 5-HIAA proved unsuitable as a biomarker due to endogenous presence. We demonstrated the methods applicability by analyzing 23 forensic blood samples, with PSG quantified in nearly all cases, even when psilocin was below LOQ. In conclusion, PSG is a reliable and specific marker of psilocybin use and its integration into routine forensic workflows could substantially improve detection reliability.

## Supplementary Material

bkag015_Supplementary_Data

## Data Availability

The data underlying this article are available in the article and in its online supplementary material.
