# Supplementary material for: Psilocin glucuronide in whole blood: a stable and useful biomarker of psilocybin intake
Source: J Anal Toxicol. 2026 Feb 22;50(4):bkag015. doi: 10.1093/jat/bkag015 (PMC13197576; doi:10.1093/jat/bkag015)
Supplement: bkag015_Supplementary_Data [file bkag015_supplementary_data.zip › jat-25-4698-File006.docx]

# Psilocin glucuronide in whole blood: a stable and useful biomarker of psilocybin intake

# Short title: Psilocin glucuronide in whole blood

## **SUPPL. 1 Method validation**

We validated the method according to AAFS guidelines [38], covering calibration model, precision, bias, LOD, LOQ, interferences, recovery, matrix effects, carry-over, and dilution integrity.

### **Calibration model**

We assessed the calibration model based on five analyses of seven calibrators with one replicate per concentration level prepared in human whole blood. Weighted calibration curves (1/x) were selected after comparison with alternative models and were created and evaluated using Masslynx™ 4.1 software (Waters). We considered the calibration curves acceptable if the correlation coefficient (R^2^) and residuals were ≥ 0.99 and ≤ ± 20 %, respectively.

### **Precision and accuracy**

Within-run precision as well as between-run precision and accuracy were assessed using five to six analyses of six QC sample concentrations prepared in human whole blood (n=3 per conc. level). We reported accuracy as bias, calculated as the percent deviation between the measured and nominal concentrations. Precision was presented as the coefficient of variation (% CV). We determined within run-precision separately for each analysis, reported the largest % CV, and considered ≤ ± 20 % acceptable. Between-run precision and accuracy were calculated for all analyses collectively and considered acceptable at a % CV and bias ≤ ± 20 %.

### **Limits of detection and quantification**

LOD and LOQ were determined based on precision and accuracy experiments. LOD was defined as the lowest QC sample concentration with a signal to noise ratio (S/N) ≥ 3.3 while LOQ was the lowest QC sample concentration where the S/N ≥ 10 for the quantifier ion, intermediate precision and accuracy were ≤ ± 20 %, and the quantifier/qualifier ion ratio deviation was within ± 20 %.

### **Recovery and matrix effects**

We assessed analyte recovery and matrix effects (ME) by analyzing four sets of samples fortified with analytes at 30 and 400 nM. Human whole blood from five sources was fortified with analytes either before sample preparation (set 1, n=5 per concentration level) or after protein precipitation and filtration (set 2, n=5 per concentration level). In set 3, blood from ten sources was fortified after protein precipitation and evaporation to dryness (n=10 per concentration level). Set 4 consisted of reconstitution solution fortified with analytes (n=5 per concentration level). Sets 1–3 were reconstituted in the same solution following evaporation and ISs were added to all four samples sets.

Recovery was determined as the ratio between the peak height of the analyte added before sample preparation (set 1) to the peak height after protein precipitation and filtration (set 2), and to the peak height after protein precipitation and evaporation to dryness (set 3). ME was calculated as the ratio of the peak height of samples fortified after sample preparation (set 3) to the peak height of analytes in fortified reconstitution solution (set 4), as described by Matuzewski et al. [39]. We considered ME acceptable in the range of 80–120 %.

### **Interference studies**

We examined matrix interferences by analysis of blood from ten different sources. Interferences from the isotope labeled ISs, which may contain traces of the unlabeled analyte, were examined by analysis of nine blank blood samples fortified with IS. Interferences from other commonly encountered drugs and pharmaceuticals were evaluated by analysis of neat standards (120–3000 nM). The following compounds were analyzed: alprazolam, amphetamine, benzoylecgonine, buprenorphine, clonazepam, codeine, cocaine, diazepam, 3,4-methylenedioxymethamphetamine (MDMA), methadone, methamphetamine, morphine, N-desmethyldiazepam, nitrazepam, oxazepam, oxycodone, phosphatidylethanol (PEth, 16:0/18:1), tetrahydrocannabinol (THC), tramadol, zolpidem, and zopiclone.

### **Processed sample stability**

We evaluated analyte stability in extracts prepared immediately after fortification of whole blood. Processed sample stability was examined for up to three days in the autosampler (10 °C), 24 hours at room temperature and one week at -20 °C. We considered analytes with a deviation from the initial concentration ≤ ± 20 % as stable.

### **Carry-over**

We evaluated carry-over by injecting three blank whole blood samples after the highest calibrator (500 nM). Carry-over was considered significant if the blank samples showed peak heights greater than 10 % of the peak height at the LOQ.

### **Dilution integrity**

We assessed dilution integrity by analyzing whole blood samples fortified with analyte at 760 nM, followed by dilutions of 1:1 and 1:4 (n=3 per dilution ratio), across five to six analyses. Within-run precision as well as between-run precision and accuracy were calculated for the diluted blood samples. We considered the dilution integrity acceptable at a % CV and bias ≤ ± 20 %.

## **SUPPL. 2 Effect of light exposure on analyte and internal standard stability in samples stored in light and darkness for three hours**

|  | **Peak Height (average)** | | | | **Change in light vs darkness (%)** |
| --- | --- | --- | --- | --- | --- |
| **Analyte** | **Darkness** | **RSD (%)** | **Light** | **RSD (%)** |  |
| Psilocin | 1973366 | 3.0 | 2004259 | 1.3 | 1.6 |
| PSG | 409241 | 2.0 | 416533 | 5.1 | 1.8 |
| Bufotenine | 851597 | 2.7 | 825443 | 1.2 | -3.1 |
| 5-HIAA | 162875 | 32 | 139593 | 5.6 | -14 |
| Psilocin-d_6_ | 386074 | 5.3 | 386730 | 3.9 | 0.2 |
| Buprenorphine glucuronide-d_4_ | 21844 | 3.0 | 21714 | 1.6 | -0.6 |
|  |  |  |  |  |  |
| 5-HIAA-d_5_ | 12293 | 27 | 10135 | 14 | -18 |

The internal standards of bufotenine and PSG were not included since they were purchased after the experiment took place

## **SUPPL. 3 Stability of analytes and internal standards under varying evaporation conditions (temperature and time)**

| **Analyte** | **Temperature** | **Peak Height, t=0** | **Normalized Stability^a^** | **RSD (%)** | **Peak Height, t=+20 min** | **RSD (%)** | **Change over 20 min (%) ^b^** |
| --- | --- | --- | --- | --- | --- | --- | --- |
| Psilocin | 30°C | 130685 | 34 | 53 | 87800 | 39 | -33 |
|  | 40°C | 379712 | 100 | 71 | 32894 | 36 | -91 |
|  | 50°C | 33394 | 9 | 195 | 21173 | 50 | -37 |
|  | 60°C | 50199 | 13 | 36 | 20394 | 48 | -59 |
| PSG | 30°C | 10681904 | 85 | 15 | 9915745 | 20 | -7 |
|  | 40°C | 9499213 | 76 | 8 | 9994370 | 23 | 5 |
|  | 50°C | 10415838 | 83 | 7 | 9803196 | 3 | -9 |
|  | 60°C | 12522807 | 100 | 9 | 9885044 | 26 | -21 |
| Bufotenin | 30°C | 16495245 | 97 | 10 | 13845387 | 32 | -16 |
|  | 40°C | 14987811 | 88 | 14 | 10628359 | 20 | -29 |
|  | 50°C | 11611483 | 68 | 24 | 8121679 | 12 | -30 |
|  | 60°C | 17017159 | 100 | 16 | 7475271 | 27 | -56 |
| 5-HIAA | 30°C | 1201043 | 74 | 18 | 995686 | 34 | -17 |
|  | 40°C | 1300949 | 80 | 19 | 742068 | 18 | -43 |
|  | 50°C | 908120 | 56 | 43 | 576352 | 18 | -37 |
|  | 60°C | 1622981 | 100 | 22 | 511408 | 18 | -68 |
| Psilocin-d_6_ | 30°C | 23253 | 35 | 48 | 15167 | 44 | -35 |
|  | 40°C | 65624 | 100 | 67 | 5835 | 35 | -91 |
|  | 50°C | 4406 | 7 | 196 | 3263 | 55 | -26 |
|  | 60°C | 6312 | 10 | 35 | 2155 | 97 | -66 |
| 5-HIAA-d_5_ | 30°C | 64178 | 73 | 26 | 54024 | 36 | -16 |
|  | 40°C | 76701 | 87 | 23 | 42805 | 17 | -44 |
|  | 50°C | 50202 | 57 | 41 | 29361 | 19 | -42 |
|  | 60°C | 88338 | 100 | 19 | 32843 | 22 | -63 |

^a^ (%) is normalized to the temperature with the highest peak height (set to 100 %), ^b^ Relative stability expressed as percentage change in peak height between t = 0 and t = 20 min. The internal standards of bufotenine and PSG were not included since they were not available in our laboratory at the time of the experiment.

## **SUPPL. 4 MRM chromatograms of the analytes at the LOD**

**
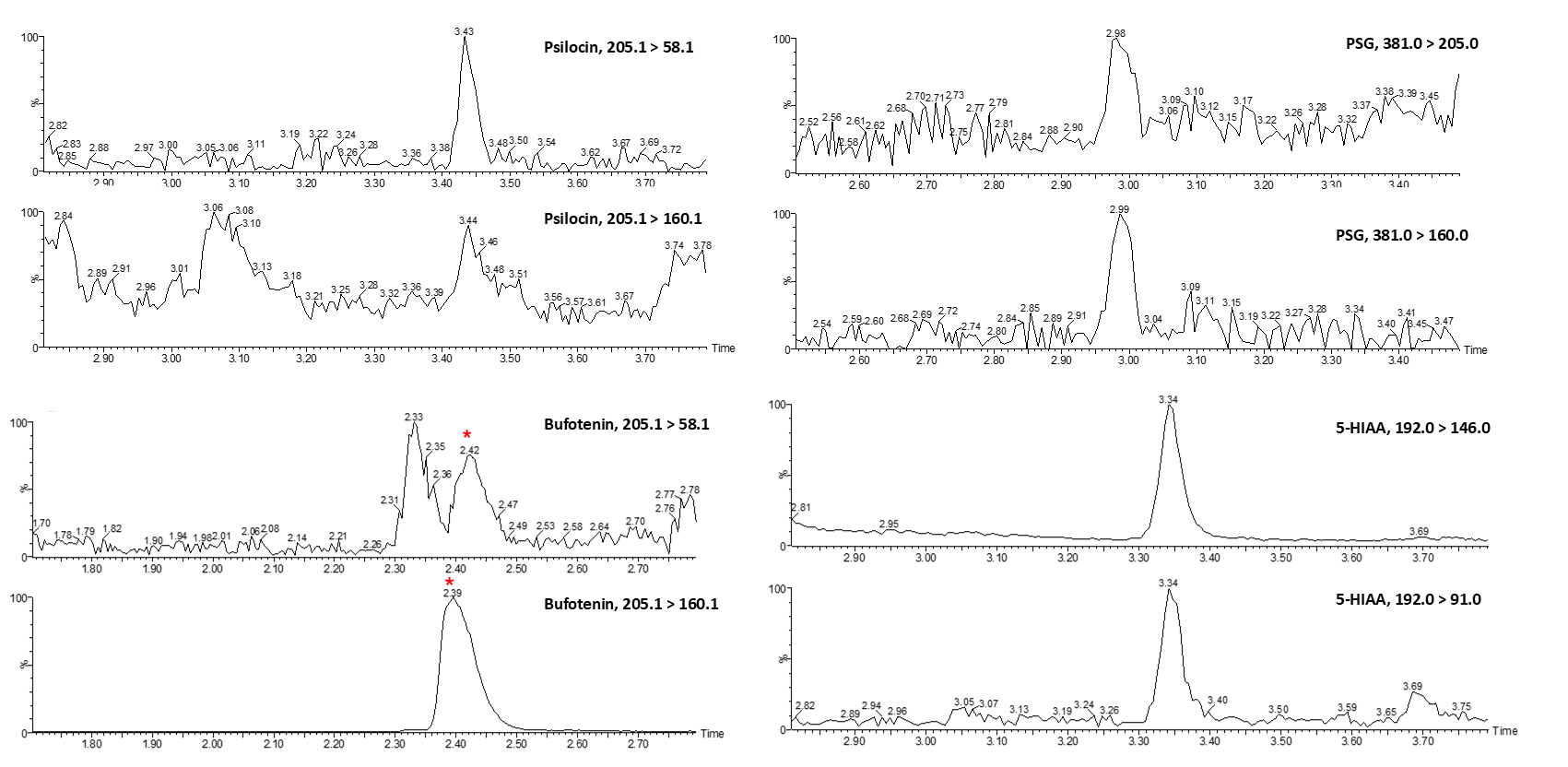
*Interference from the whole blood matrix**

## **SUPPL. 5 MRM chromatograms of the analytes at the LOQ**

**
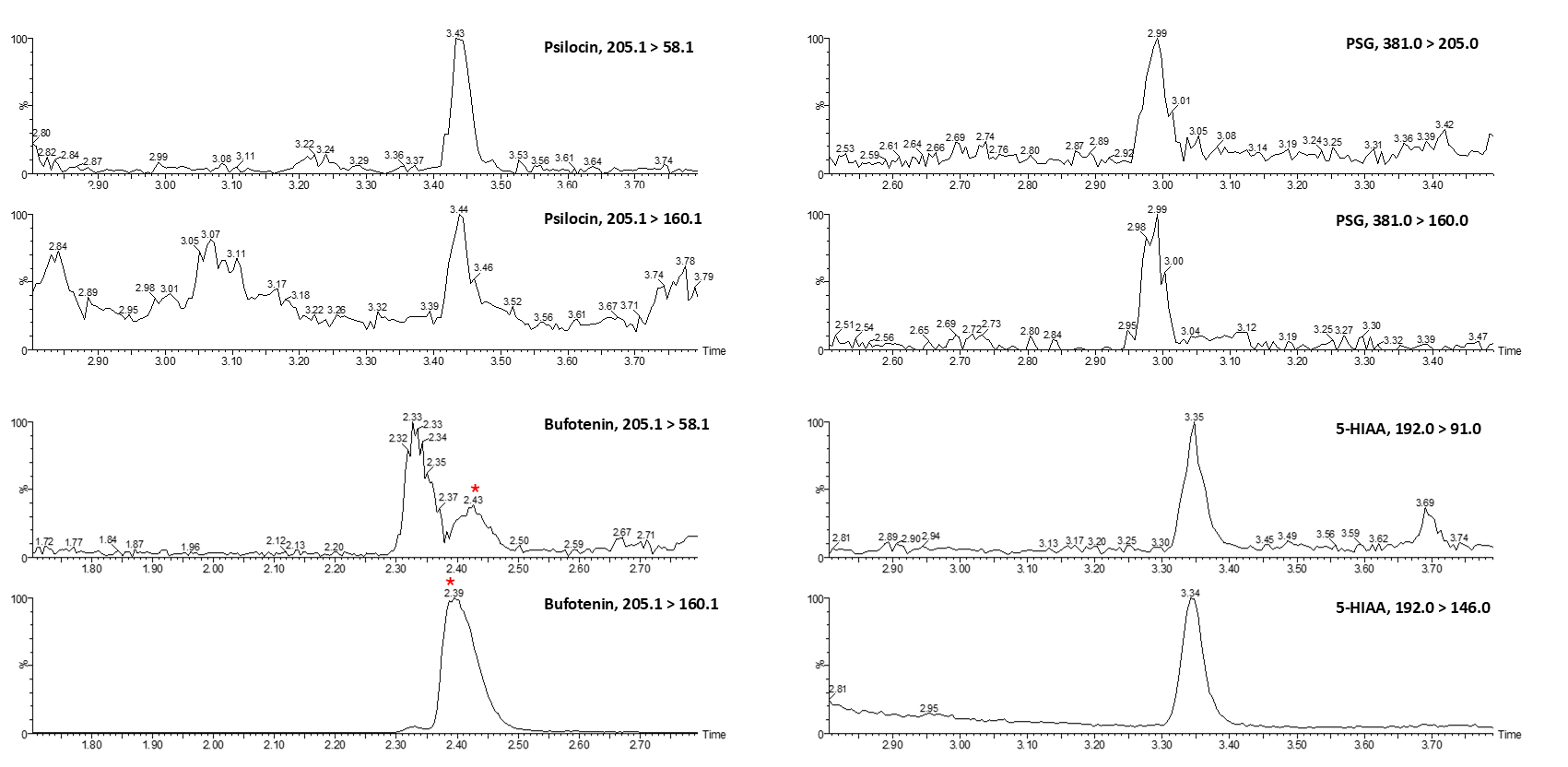
*Interference from the whole blood matrix**

## **SUPPL. 6 Stability of analytes in whole blood at different storage conditions and after 3 freeze/thaw cycles^a^**

|  | **Analyte** | **Psilocin** | | **PSG** | | | **Bufotenin** | | **5-HIAA^b^** | |
| --- | --- | --- | --- | --- | --- | --- | --- | --- | --- | --- |
| Nominal concentration (nM)^c^ |  | 30 | 400 | 30 | | 399 | 30 | 401 | 30 | 401 |
| Calculated concentration (nM) | Day 0 | 32 (4.5) | 439 (1.9) | | 29 (3.0) | 432 (4.3) | 29 (3.4) | 403 (4.3) | 90 (51) | 514 (8.0) |
| Stability in whole blood at room temperature, Bias (%) | Day 1 | -46 (10) | -66 (17) | | -16 (3.5) | -0.9 (6.9) | -3.3 (2.5) | -12 (4.8) | 90 (46) | -8.0 (12) |
|  | Day 3 | -88 (22) | -99 (29) | | -5.0 (2.8) | -7.1 (4.1) | -0.5 (2.0) | -20 (3.5) | 77 (43) | -25 (2.3) |
|  | Day 7 | -100 | -100 (2.0) | | 2.7 (1.1) | 0.9 (2.4) | -17 (2.9) | -34 (4.4) | 42 (44) | -48 (12) |
|  | Day 14 | -100 | -100 | | 0.3 (6.6) | -0.5 (4.0) | -20 (6.8) | -45 (9.5) | -4.0 (52) | -59 (4.7) |
| Stability in whole blood at 4 °C, Bias (%) | Day 1 | -76 (63) | -66 (33) | | N. M | N. M | N. M | N. M | N. M | N. M |
|  | Day 3 | -100 | -94 (104) | | N. M | N. M | N. M | N. M | N. M | N. M |
|  | Day 7 | -100 | -100 (4.6) | | -4.0 (3.2) | -1.5 (0.6) | -0.2 (3.2) | -17 (6.7) | 80 (31) | -22 (9.3) |
|  | Day 14 | -67 (18) | -99 (86) | | 0.3 (6.8) | 2.0 (4.7) | -0.6 (7.4) | -32 (11) | 94 (50) | -24 (7.0) |
| Stability in whole blood at -20 °C, Bias (%) | Day 7 | -32 (7.9) | -24 (5.5) | | -6.8 (1.7) | -2.7 (8.6) | -0.8 (1.6) | 5.3 (1.7) | 59 (37) | -7.7 (16) |
|  | Day 14 | -27 (26) | -43 (14) | | -4.1 (5.8) | -2.3 (4.2) | 5.0 (5.0) | -2.9 (3.8) | 96 (48) | -0.4 (12) |
|  | Day 30 | -51 (55) | -49 (26) | | -4.2 (5.1) | -8.3 (4.6) | -22 (17) | -11 (5.3) | -51 (139) | -29 (12) |
|  | Month 3 | -100 | -91 (82) | | -10 (4.2) | -10 (11) | -52 (5.4) | -27 (10) | 343 (86) | -27 (16) |
|  | Year 1^d^ | -100 | -100 | | 13 | 3.2 | N.Q | N.Q | N.Q | N.Q |
| Freeze/thaw stability, Bias (%) | Cycle 1 | -15 (8.8) | -3.5 (2.5) | -5.5 (4.4) | | 9.9 (5.0) | -1.1 (9.2) | 12 (2.2) | 60 (47) | 2.6 (17) |
|  | Cycle 2 | -18 (2.9) | -15 (5.8) | -8.4 (4.4) | | -3.0 (4.9) | 5.8 (2.6) | -1.3 (6.3) | 88 (43) | -4.6 (13) |
|  | Cycle 3 | -32 (16) | -30 (6.3) | 2.7 (4.1) | | 3.6 (4.3) | 1.7 (4.2) | 3.3 (4.8) | 71 (44) | -6.8 (15) |

^a^ RSD (%) in parentheses. N=3 for all conditions and concentrations except 1-year stability at -20°C (N=1), ^b^Endogenous, ^c^The target fortification level was 400 nM; the nominal concentrations of 399 and 401 nM correspond to the actual concentrations prepared, ^d^ no RSD since n=1, N.M.: Not measured.

## **SUPPL. 7 Stability of analytes in extracted samples stored in autosampler and room temperature^a^**

|  | **Analyte** | **Psilocin** | | **PSG** | | **Bufotenine** | | **5-HIAA** | |
| --- | --- | --- | --- | --- | --- | --- | --- | --- | --- |
| Nominal concentration (nM)^b^ |  | 30 | 400 | 30 | 399 | 30 | 401 | 30 | 401 |
| Calculated concentration (nM) | Day 0 | 32 (2.1) | 430 (2.2) | 32 (5.6) | 435 (2.2) | 33 (3.2) | 422 (1.1) | 27 (16) | 428 (4.9) |
| Stability in autosampler, Bias (%) | Day 1 | 7.7 (6.0) | 7.3 (2.3) | 12 (3.9) | 12 (4.6) | 14 (3.9) | 9.7 (2.5) | -22 (17) | 4.5 (6.6) |
|  | Day 2 | 5.2 (6.4) | 1.8 (4.9) | 5.0 (7.1) | 5.0 (3.8) | 1.7 (5.2) | 1.1 (4.9) | 11 (19) | -18 (3.8) |
|  | Day 3 | 6.0 (3.0) | 7.4 (4.3) | -4.4 (7.6) | -2.9 (3.6) | 12 (11) | 8.7 (12) | 1.1 (27) | 3.7 (4.0) |
| Stability at Room temperature, Bias (%) | Day 1 | 3.9 (8.7) | 8.3 (2.3) | 5.0 (2.0) | 13 (4.6) | 13 (5.2) | 13 (2.5) | -55 (26) | 1.3 (6.6) |
| Stability at -20 °C, Bias (%) | Day 7 | 10 (4.6) | 3.3 (4.9) | -0.8 (2.3) | -1.6 (3.8) | 4.5 (2.4) | 0 (4.9) | 46 (11) | -0.9 (3.8) |

^a^RSD (%) is provided in parentheses, ^b^The target fortification level was 400 nM; the nominal concentrations of 399 and 401 nM correspond to the actual concentrations prepared
